# Obesity and Cardiovascular Risk: Variations in Visfatin Gene Can Modify the Obesity Associated Cardiovascular Risk. Results from the Segovia Population Based-Study. Spain

**DOI:** 10.1371/journal.pone.0153976

**Published:** 2016-05-11

**Authors:** María Teresa Martínez Larrad, Arturo Corbatón Anchuelo, Cristina Fernández Pérez, Milagros Pérez Barba, Yera Lazcano Redondo, Manuel Serrano Ríos, Segovia Insulin Resistance Study Group (SIRSG)

**Affiliations:** 1 Spanish Biomedical Research Centre in Diabetes and Associated Metabolic Disorders (CIBERDEM), Madrid, Spain; 2 Instituto de Investigación Sanitaria del Hospital Clínico San Carlos (IdISSC-HCSC), Madrid, Spain; GDC, GERMANY

## Abstract

**Objectives:**

Our aim was to investigate if genetic variations in the visfatin gene (SNPs rs7789066/ rs11977021/rs4730153) could modify the cardiovascular-risk (CV-risk) despite the metabolic phenotype (obesity and glucose tolerance). In addition, we investigated the relationship between insulin sensitivity and variations in visfatin gene.

**Material and Methods:**

A population-based study in rural and urban areas of the Province of Segovia, Spain, was carried out in the period of 2001–2003 years. A total of 587 individuals were included, 25.4% subjects were defined as obese (BMI ≥30 Kg/m^2^).

**Results:**

Plasma visfatin levels were significantly higher in obese subjects with DM2 than in other categories of glucose tolerance. The genotype AA of the rs4730153 SNP was significantly associated with fasting glucose, fasting insulin and HOMA-IR (Homeostasis model assessment-insulin resistance) after adjustment for gender, age, BMI and waist circumference.

The obese individuals carrying the CC genotype of the rs11977021 SNP showed higher circulating levels of fasting proinsulin after adjustment for the same variables.

The genotype AA of the rs4730153 SNP seems to be protective from CV-risk either estimated by Framingham or SCORE charts in general population; and in obese and non-obese individuals. No associations with CV-risk were observed for other studied SNPs (rs11977021/rs7789066).

**Conclusions:**

In summary, this is the first study which concludes that the genotype AA of the rs4730153 SNP appear to protect against CV-risk in obese and non–obese individuals, estimated by Framingham and SCORE charts. Our results confirm that the different polymorphisms in the visfatin gene might be influencing the glucose homeostasis in obese individuals.

## Introduction

Nowadays obesity has become epidemic in developed and even in developing countries worldwide [1].This disease is characterized by low-grade systemic inflammation [2]. Regarding this affirmation, it is fully accepted that the adipose tissue is a metabolically active endocrine organ secreting a variety of bioactive substances known as adipokines, that could act as functional links between energy balance and insulin resistance [3, 4]. Visfatin is a recently described adipocytokine, also known as pre-B cell colony-enhancing factor (PBEF) or nicotinamide phosphorybosil transferase (NAMPT)[5].NAMPT was cloned by Samal and his colleagues in 1994 from activated human peripheral blood lymphocytes during their attempt to discover new factors for the earliest events in B-cell development [6]. This protein of 52–55 KDa, was described as a growth factor for early B cells called pre-B cell colony–enhancing factor 1 (PBEF1). Recently, it has been identified as an intracellular enzyme and called nicotinamide 5-phosphoribosyl-1-pyrophosphate transferase (NAMPT) that catalyzes the rate-limiting step in nicotinamide adenine dinucleotide (NAD) biosynthesis and mediates the conversion of nicotinamide to nicotinamide mononucleotide [7–9].It has been proposed that this adipose derived hormone exerts insulin mimicking effects and play a positive role in attenuating insulin resistance. However, the precise mechanisms underlying the beneficial effects of visfatin on insulin sensitivity remain largely unknown [3]. It has been suggested that visfatin might have both endocrine [7,10] and paracrine effects [11], mostly related to obesity and insulin sensitivity although there are important discrepancies in the literature[12]. As regards to obesity, Fukuhara et al. reported that, in adipose tissue, visfatin is predominantly expressed by visceral fat as estimated by abdominal computed tomography; and also its circulating levels correlate with the amount of visceral fat in both humans and mice [7]. However, these observations were not confirmed by other authors [13–15]. Furthermore, the significance of the correlation between visfatin levels and body mass index (BMI) is still unclear [15–17] probably due to deficiencies in the specificity of the applied immunoassays [18].

As it relates to the impact of visfatin on insulin sensitivity, Fukuhara *et al* reported that visfatin binds to and activates the insulin receptor explaining the reported metabolic effect of this citokyne on peripheral organs [7]. Recently, these authors retracted from the original paper [19], being this hypothesis currently under debate [12]. Moreover, other groups have reported on the presumed insulin mimetic effects of visfatin in osteoblasts and cultured mesangial cells [10,20]. Visfatin is a proinflammatory mediator and might participate in a variety of inflammatory conditions, such as autoimmune diseases and adipose tissue inflammation-induced insulin resistance [21].

The human visfatin gene is located on chromosome 7q22.3 and includes 11 exons encompassing 34.7 kb. Interestingly, this region appears to be linked to metabolic syndrome–related phenotypes in several populations including non diabetic Mexican-Americans [22], Mexicans–American families [23] and hypertensive Hispanic families [24] according to multipoint variance component analysis. Our aim was to investigate if genetic variations in the visfatin gene could be associated with obesity, glucose tolerance (Diabetes Mellitus Type 2) and cardiovascular (CV)risk–related alterations on a large sample of a population–based study in Spain.

## Methods

### Participants

The Segovia Study was designed as a cross-sectional, population–based survey of the prevalence of anthropometric and physiological parameters related to obesity and other components of the metabolic syndrome. It was conducted in rural and urban areas of the province of Segovia, in Central Spain. A detailed report of this study has been previously published [25]. In brief, a random sample of 2,992 men and non—pregnant women aged 35–74 years were selected from a target population of 63,417 inhabitants. A total of 587 individuals gave written consent after receiving detailed information on the purposes and the objectives of the study. The protocol was approved by the Ethics Committee of the Hospital Clínico San Carlos in Madrid.

### Anthropometrical and biochemical measurements

The BMI and waist circumference (WC) were used as estimations of total body fat mass and visceral obesity, respectively. Those individuals with a BMI higher than 30 kg/m^2^ were classified as obese. Systolic and diastolic blood pressures were measured three times in a seated position after 10 min of rest to the nearest even digit using a random-zero sphygmomanometer. 20 ml of blood were obtained from an antecubital vein without compression after about a ten-hour overnight period. Plasma glucose was determined twice by a glucose-oxidase method adapted to autoanalyze (Hitachi 704, Boehringer Mannheim, Germany). An oral glucose tolerance test (OGTT) was performed following the criteria of the American Diabetes Association [26]. The categories of glucose values were as follows: 1) normoglycaemia (NG): fasting plasma glucose < 100 mg/dl or 2-h postload glucose lower than 140 mg/dl; 2) impaired fasting glucose (IFG): fasting plasma glucose levels ≥ 100 mg/dl but <126 mg/dl and 2h postload glucose lower than 140 mg/dl; 3) impaired glucose tolerance (IGT): 2-h postload glucose between 140 and 200 mg/dl; 4) diabetes: fasting plasma glucose levels ≥ 126 mg/dl or 2-h postload glucose ≥ 200 mg/dl. Total serum cholesterol, triglycerides and high-density lipoprotein cholesterol (HDL-C) were determined by enzymatic methods using commercial kits (Boehringer Mannheim, Germany). Low-density lipoprotein cholesterol (LDL-C) was calculated by the Friedewald formula. Plasma visfatin concentrations were measured with a human visfatin (COOH-terminal) enzyme immunometric assay (Phoenix, Belmont, CA). The minimum detectable concentration was 2.3 ng/ml and the intra / inter-assay variation´s coefficient <5% and <14% respectively. Insulin, proinsulin, leptin and adiponectin serum concentrations were determined by highly specific / sensitive RIAs (Linco Research Inc., St LouisMO, USA). Insulin resistance (IR) was estimated by the homeostasis model assessment (HOMA-IR) method according to the formula: insulin (μU/ml) x glucose (mmol/l) / 22.5 [27], using a cut-off point for HOMA-IR ≥ 3.8 as described in the Spanish population [28].

To estimate high CV risk we chose: a) Framingham chart by D´Agostino et al [29] in which the top sex-specific quintiles of predicted CVD risk identifies ~ 48% of men and 58% of women who experienced a first CVD event on follow-up (sensitivity) and proportions of men and women without events who were not in the top quintile of risk were 85% and 83% respectively (specificity); b) SCORE Cholesterol risk chart for low risk regions [30]: the 5% threshold has a 35% sensitivity and 88% specificity and the ROC area is 0.74 (95%CI 0.72–0.76). A CVR ≥20% according to the Framingham chart is associated with high risk of cardiovascular morbidity and a CVR ≥5% with the SCORE chart indicates high risk of cardiovascular mortality.

### Genotyping

Three previously described (http://www.ncbi.nlm.nih.gov/SNP) visfatin gene variants were genotyped. In order to select the polymorphic sites in the NAMPT gene that would be genotyped in our sample, we used information from the HapMap database. Previous studies have reported association of NAMPT variants with homeostasis glucose, fasting insulin [17, 31–33] and metabolic disorders [34]. Among these, the SNPs rs7789066 and rs11977021 reside within the promoter region and the SNP rs4730153 is located in an intronic region. DNA was extracted from venous blood using a kit by Qiagen. Genotyping was performed using the ABI PRISM 7900 Sequence Detection System by TaqMan for allelic discrimination assay (Applied Biosystems). Genotyping was done using Assays–on Demand, and SNP Genotyping Products (Applied Biosystems). The reaction was amplified on a GeneAmp polymerase chain reaction system: cycling conditions were as follows: an enzyme heat activation step at 95°C for 10 min, 40 two-step amplification cycles at 95°C for 15 second for denaturation and a last heating step at 60°C for 1 min for annealing and extension. To assess genotyping reproducibility, a random ~20% selection of the samples was genotyped, again in three SNPs with 100% concordance.

### Statistical Analysis

Genotypic and allelic frequencies were analysed as categorical variables. Hardy-Weinberg equilibrium was computed to the expected genotype distribution. Triglycerides, fasting insulin, HOMA-IR, and leptin values were log-transformed because of their skewed distributions. The one-way analysis of variance (ANOVA) was used to compare continuous variables expressed as the mean ± standard deviation (S.D.), while categorical variables were compared using the Chi-squared test. Successive multiple linear regression analyses, adjusted for potential confusion factors, were carried out to asses the effect of clinical variables and genetic variants on glucose tolerance and obesity–related alterations. The null hypothesis was rejected in each statistical test when the p value was less than 0.05. The statistical analysis was performed using the Windows SPSS version 15.0 software (SPSS, Inc, Chicago, IL, USA).

## Results

### Segovia study sample characteristics

The clinical characteristics of the studied population are shown in Tables 1 and 2. One hundred and forty-nine participants were obese, and the remaining ones (438) were non–obese. Statistical significant differences were found for most of the clinical parameters when comparing obese against normal weight subjects, although only 2 hours glucose, fasting insulin and proinsulin, 2 hours insulin and proinsulin, HOMA-IR and fasting leptin could be considered of different significant clinical relevance between groups. Fasting visfatin and adiponectin were similar in both categories subjects (Table 1). Women showed lower mean values of WC, triglycerides, fasting glucose, HOMA-IR, fasting proinsulin and proinsulin 2 hours than men. However, fasting HDL-C, leptin and adiponectin levels were higher in women than men (Table 2).

**Table 1:** Segovia Study Sample Characteristics: non-obese vs. obese subjects.

	Non-Obese(N = 438) X(SD)	Obese Subjects(N = 149)X(SD)	p
Age (years)	54 (12)	59 (12)	<0.001
BMI (kg/m^2^)	25.73 (2.63)	32.71 (2.84)	<0.001
Waist circumference (cm)	86.73 (9.91)	100.86 (7.84)	<0.001
SBP (mm Hg)	123 (17)	132 (18)	<0.001
DBP (mm Hg)	77 (9)	82 (8)	<0.001
Fasting glucose (mg/dl)	89 (29)	95 (24)	0.043
2h glucose (mg/dl)	109 (38)	127 (51)	<0.001
FastingProinsulin (pmol/L)	8.93 (5.94)	14.25 (11.96)	<0.001
2h Proinsulin (pmol/L)	52.47 (40.86)	68.54 (39.22)	<0.001
Fasting insulin (μU/ml)	12.18 (7.42)	17.40 (11.43)	<0.001
2h insulin (μU/ml)	73.87 (62.65)	97.80 (86.33)	0.001
HOMA index	2.73 (2.23)	4.18 (3.54)	<0.001
Cholesterol (mg/dl)	210 (39)	221 (40)	0.003
Triglycerides (mg/dl)	91 (54)	124 (77)	< 0.001
HDL-cholesterol (mg/dl)	63 (19)	56 (16)	< 0.001
LDL-cholesterol (mg/dl)	129 (34)	141 (36)	< 0.001
Fasting Visfatin (ng/dl)	9.74 (3.89)	10.12 (4.23)	0.296
Fasting leptin (ng/ml)	9.24 (6.60)	19.06 (11.67)	< 0.001
Adiponectin (μg/ml)	10.86 (5.24)	10.55 (5.02)	0.541

BMI, body mass index; SBP, systolic blood pressure; DBP, diastolic blood pressure. Continuous variables were compared by ANOVA and results are mean (standard deviation) S.D.

**Table 2:** Segovia Study Sample characteristics by sex.

	Males(N = 287)X(SD)	Females(N = 330)X(SD)	p
Age (years)	55 (12)	55 (12)	0.771
BMI (kg/m^2^)	27.44 (3.41)	27.59 (4.62)	0.780
Waist circumference (cm)	95.67 (9.27)	85.60 (11)	< 0.001
SBP (mm Hg)	126.27 (16.89)	124.49 (18.90)	0.278
DBP (mm Hg)	78.76 (8.57)	77.67 (8.72)	0.168
Fasting glucose (mg/dl)	93.09 (24.63)	88.51 (25.69)	0.047
2h glucose (mg/dl)	114.34 (45.07)	114.52(41.43)	0.966
Fasting Proinsulin (pmol/L)	11.52 (9.53)	9.32 (7.24)	0.005
2h Proinsulin (pmol/L)	64.03 (45.21)	50.59 (37.02)	0.001
Fasting insulin (μU/ml)	13.48 (7.52)	12.64 (8.59)	0.250
2h insulin (μU/ml)	78.12 (69.02)	79.50 (66.01)	0.840
HOMA index	3.12 (2.19)	2.84 (2.67)	0.016
Cholesterol (mg/dl)	213 (39)	221 (40)	0.538
Triglycerides (mg/dl)	115.53 (77)	85.02 (42)	< 0.001
HDL-cholesterol (mg/dl)	56 (17)	65 (18)	< 0.001
LDL-cholesterol (mg/dl)	133.96 (35)	129.11 (33)	0.131
Fasting Visfatin (ng/dl)	9.80 (3.69)	10.41 (4.23)	0.234
Fasting leptin (ng/ml)	5.83 (3.57)	16.66 (10.05)	< 0.001
Adiponectin (μg/ml)	7.98 (3.60)	13.24 (4.97)	<0.001

BMI, body mass index; SBP, systolic blood pressure; DBP, diastolic blood pressure. Continuous variables were compared by ANOVA and results are mean (standard deviation) S.D.

### Circulating visfatin levels and clinical parameters

Plasma visfatin levels were significantly higher in obese subjects with type 2 diabetes than in other categories of glucose tolerance: Normoglycaemia (NG): 9.69±3.77 ng/ml, prediabetes: impaired fasting glucose/impaired glucose tolerance (IFG/IGT): 9.61± 2.72 ng/ml, and Diabetes Mellitus (DM): 13.48± 9.66ng/ml; global p = 0.018, NG vs DM p = 0.007; IFG/IGT vs DM p = 0.016. This association between elevated visfatin levels and type 2 diabetes mellitus was confirmed by a lineal regression analysis adjusted for gender, age, BMI, and WC (β = 0.064, 95%CI: 1.002–1.135, p = 0.021). No evidence of that association was found in non obese subjects (β = 1.03, 95% CI 0.89–1.19, p = 0.067).

### Genotype distributions and association study

Genotype distributions of the analyzed SNPs are shown in Table 3. None of theSNPs deviated significantly from the Hardy-Weinberg, and our genotype distributions are similar to those reported in the NCBI (National Center for Biotechnology Information) for European populations (http://www.ncbi.nlm.nih.gov/entrez/). The analysed SNPs were in Hardy–Weinberg´s equilibrium (data not shown). In order to detect some possible associations between these genetic variants of the visfatin gene, obesity and other glucose tolerance status–related disturbances, the association study was performed in two groups of obese and non–obese individuals. Evidence of some associations with insulin sensitivity–related parameters was detected in the subgroup of obese participants. The significant associations found by ANOVA were confirmed by linear regression analysis showing that, in obese participants, the genotype AA of the rs4730153 SNP was significantly associated with fasting glucose (β = 10.84, p = 0.040), fasting insulin (β = 9.004, p = 0.003) and HOMA-IR (β = 0.173, p = 0.031) after adjustment for gender, age, BMI and WC. We performed the same analysis excluding diabetic subjects and the results were similar. On the other hand, the obese individuals carrying the CC genotype of the rs11977021 SNP showed higher circulating levels of fasting proinsulin (β = 4.231, p = 0.029) after adjustment for the same variables. No evidence of these associations was found in the non-obese subgroup or between the analysed genotypes and the other metabolic parameters (data not shown).

**Table 3:** Genotype distributions.

SNP	Variant type (bp position)	Genotype	Genotype distribution	Allelic distribution	
rs11977021	Promoter(-3186)	CC	55.7 (n = 326)	Allele C	75.4%
		CT	39.5 (n = 232)	Allele T	24.6%
		TT	4.8 (n = 29)		
rs7789066	Promoter(-2423)	AA	86.3 (n = 507)	Allele A	92.8%
		AG	13.0 (n = 76)	Allele G	7.2%
		GG	0.7 (n = 4)		
rs4730153	Intron 6(21179)	AA	17.0 (n = 100)	Allele A	41.6%
		AG	49.1 (n = 288)	Allele G	58.4%
		GG	33.9 (n = 199)		

### Cardiovascular risk

The genotype AA of the rs4730153 SNP offers protection against CV risk either estimated by Framingham or SCORE charts in general population as well as in obese and non-obese (Table 4).No associations with CV risk were observed for the other studied SNPs (rs11977021,rs7789066).

**Table 4:** Cardiovascular risk according to SNP: rs4730143.

SNP	rs4730153	FRAMINGHAM				SCORE			
		< 20%		≥ 20%		< 5%		≥ 5%	
	Genotype	n	%	n	%	n	%	n	%
	GG	149	79.4	39	20.6	164	87.3	24	12.7
	GA	241	85.6	41	14.4	256	90.8	26	9.2
	AA	105	90	12	10	112	95.6	5	4.4
		p = 0.019				p = 0.031			

## Discussion

Most recently it has been demonstrated that the systemic visfatin-mediated nicotinamide adenine dinucleotide synthesis is necessary for β cell function [35]. In this study, we performed a genetic association analysis to investigate whether the visfatin gene is a candidate gene influencing glucose tolerance, obesity and CV risk. The results here reported show for the first time, that in obese adult population, the SNP rs4730153 is associated with insulin resistance as estimated by HOMA-IR regardless of BMI, WC, age and gender. Moreover, another SNP, namely rs11977021 is independently associated with serum proinsulin levels. On the other hand the genotype AA of the rs4730153 SNP seemed to be protective for CV risk in either Framingham or SCORE in general population as well as in obese and non-obese.

As for the significance of circulating visfatin levels, several authors [13, 14, 36] have reported higher circulating visfatin levels in type 2 diabetic subjects than in non diabetic individuals from Caucasian and non–Caucasian populations, though it was unclear if this association was primarily related to obesity. Moreover, no associations between insulin sensitivity related parameters and circulating visfatin levels have been found in most of the reported studies [13, 14, 37, 38] or, when they found significance, it was lostafter adjusting for BMI [39]. Chen et al [40], in a case–control study on 120 non–obese individuals, reported higher plasma levels of circulating visfatin in patients with type 2 diabetes mellitus than in non diabetic subjects after adjustment for gender, age and BMI. Similarly, García-Fuentes et al [16] showed that plasma visfatin levels are increased in patients with severe obesity but only when it is accompanied by some degree of impaired glucose tolerance. Using the same EIA commercial kit (Phoenix Pharmaceuticals), we found that elevated visfatin levels were associated with a higher prevalence of type 2 diabetes mellitus just in obese subjects. However, the conclusions derived from our study and the above referred reports must have some limitations because of the specificity of this particular assay, as well as others currently available, has been recently questioned as the different immunoassays might detect distinct visfatin compounds [18].

On the other hand, the analysis of genetic variants in the visfatin gene might help knowing if visfatin plays an important physiological role in glucose homeostasis–related alterations [41]. We found that, in our obese subpopulation, the SNPs rs11977021 and rs4730153 are associated with glucose metabolism–related parameters regardless of BMI, WC, age and gender. As for the rs4730153 variant, the allele frequency was in agreement with that reported in other Caucasian populations [31, 42]. The homozygous subjects for the A allele had higher mean values of fasting glucose, fasting insulin and HOMA index. These associations have been previously explored in 626 subjects without type 2 diabetes mellitus [31] and in 167 obese and 508 non-obese children [41] from Germany, but no statistically significant associations were found. The physiological mechanism by which an intronic polymorphism such as the rs4730153 affects changes in glucose metabolism remains unclear. So far, there are only 2 SNPs located in the promoter region of the visfatin gene (-948C/A, rs9770242) which have been found to be associated with type 2 diabetes–related parameters [31, 32, 43], but no proof of its functionality is yet available. Of further interest is to consider that polymorphisms in the phosphoinositide 3 –kinase gene is a potential candidate gene to develop type 2 diabetes [44] also located in the chromosome region 7q22.3.

The rs11977021 had been previously associated with total and LDL cholesterol [31] but its possible association with proinsulin has not been previously analysed. We report here a significant association between the CC genotype of the rs11977021 and higher levels of fasting proinsulin. This finding might be of relevance since there is data indicating that the rs11977021 variant might be a functional polymorphism key in type 2 diabetes mellitus [31].

On the other hand, in the last years, several studies have established positive associations between enhanced circulating visfatin/NAMPT levels and atherogenic inflammatory diseases, therefore supporting a role for visfatin as a potential biomarker of cardiovascular complications associated to metabolic disorders [45–47]. In another study, Belo et al [48] explored different SNPs (rs1319501 and rs3801266) in obese children and adolescence with and without ≥ 3 traditional metabolic risk factors (MRFs). They found that the SNPs rs1319501 had no effect on plasma visfatin levels in obese and obese children with ≥ 3 MRFs as compared to lean children. In contrast, rs3801266 was associated with higher plasma visfatin concentrations in lean and obese children but not in obese children with ≥ 3 MRFs, suggesting that obesity and MRFs are more influential than genetic polymorphisms in the determination of visfatin levels in obese children. Other study in obese Chinese children and adolescents [49] found that rs4730153 GG genotype polymorphism was related to an increase in insulin sensitivity and a decrease in triglycerides in response to exercise. So previous described results focused on obesity in earlier stages of life, need to be corroborated in larger studies and the comparison to our results here reported is difficult. Finally, Dou et al.[50] suggested that NAMPT may play an important role in the development of dilated cardiomyopathy. Our study shows that visfatin genotype AA SNP rs4730153 is associated with a lower estimated CV-risk, although the precise mechanism by which the SNP influence CV-risk remains to be established.

In summary, this is the first study which concludes that the genotype AA of the rs4730153 SNP appear to protect against CV events in obese and non-obese individuals, estimated by Framingham and SCORE charts. Our results confirm that the different polymorphism in the visfatin gene might be influencing the glucose homeostasis in obese individuals. Additional genetic studies in large obese population samples and functional characterization of the genetic variants are, however, warranted to corroborate our results.

## Abbreviations

CV: risk Cardiovascular-risk
PBEF1: Pre-B cell colony–enhancing factor 1
NAMPT: nicotinamide 5-phosphoribosyl-1-pyrophosphate transferase
NAD: nicotinamide adenine dinucleotide
HOMA-IR: Homeostasis model assessment- insulin resistance
BMI: Body Mass Index
CV: cardiovascular
WC: waist circumference
OGTT: oral glucose tolerance test
NG: normoglycaemia
IFG: impaired fasting glucose
IGT: impaired glucose tolerance
HDL-C: high-density lipoprotein cholesterol
LDL-C Low: density lipoprotein cholesterol
IR: Insulin resistance
SCORE: Systematic Coronary Risk Evaluation
NCBI: National Center for Biotechnology Information
